# Clinical validation of an automatic atlas‐based segmentation tool for male pelvis CT images

**DOI:** 10.1002/acm2.13507

**Published:** 2022-01-22

**Authors:** Marta Casati, Stefano Piffer, Silvia Calusi, Livia Marrazzo, Gabriele Simontacchi, Vanessa Di Cataldo, Daniela Greto, Isacco Desideri, Marco Vernaleone, Giulio Francolini, Lorenzo Livi, Stefania Pallotta

**Affiliations:** ^1^ Medical Physics Unit Careggi University Hospital Florence Italy; ^2^ Department of Experimental and Clinical Biomedical Sciences University of Florence Florence Italy; ^3^ National Institute of Nuclear Physics (INFN) Florence Italy; ^4^ Radiation Oncology Unit Careggi University Hospital Florence Italy; ^5^ Florentine Institute of Care and Assistance (IFCA) Florence Italy

**Keywords:** auto‐contouring, CT, inter‐observer, intra‐observer, pelvis, time savings

## Abstract

**Purpose:**

This retrospective work aims to evaluate the possible impact on intra‐ and inter‐observer variability, contouring time, and contour accuracy of introducing a pelvis computed tomography (CT) auto‐segmentation tool in radiotherapy planning workflow.

**Methods:**

Tests were carried out on five structures (bladder, rectum, pelvic lymph‐nodes, and femoral heads) of six previously treated subjects, enrolling five radiation oncologists (ROs) to manually re‐contour and edit auto‐contours generated with a male pelvis CT atlas created with the commercial software MIM MAESTRO. The ROs first delineated manual contours (M). Then they modified the auto‐contours, producing automatic‐modified (AM) contours. The procedure was repeated to evaluate intra‐observer variability, producing M1, M2, AM1, and AM2 contour sets (each comprising 5 structures × 6 test patients × 5 ROs = 150 contours), for a total of 600 contours. Potential time savings was evaluated by comparing contouring and editing times. Structure contours were compared to a reference standard by means of Dice similarity coefficient (DSC) and mean distance to agreement (MDA), to assess intra‐ and inter‐observer variability. To exclude any automation bias, ROs evaluated both M and AM sets as “clinically acceptable” or “to be corrected” in a blind test.

**Results:**

Comparing AM to M sets, a significant reduction of both inter‐observer variability (*p* < 0.001) and contouring time (‐45% whole pelvis, *p* < 0.001) was obtained. Intra‐observer variability reduction was significant only for bladder and femoral heads (*p* < 0.001). The statistical test showed no significant bias.

**Conclusion:**

Our atlas‐based workflow proved to be effective for clinical practice as it can improve contour reproducibility and generate time savings. Based on these findings, institutions are encouraged to implement their auto‐segmentation method.

## 1 INTRODUCTION

In radiotherapy treatment planning, image segmentation is a time‐consuming task that exhibits great variability among radiation oncologists (ROs). Automatic approaches reduce the contouring workload, but the main challenges in computed tomography (CT) images segmentation are represented by the scarce contrast between soft tissue structures and the huge anatomical variability between patients. In the last few years, several semi‐automated or automated segmentation approaches have been proposed to support ROs in reducing contouring time.^1^, ^2^, ^3^, ^4^, ^5^ Among automated segmentation approaches, only those based on atlases^6^, ^7^, ^8^, ^9^, ^10^ or on artificial intelligence (AI)^6^, ^11^, ^12^, ^13^, ^14^, ^15^ can aim to fully automate contouring processes. Atlas‐based methods are implemented by several vendors as treatment planning system options or stand‐alone software modules. Users can take advantage of atlases provided with the software module or can create their own, based on their patients’ images and local contouring guidelines. However, all automatic contours need a careful review and a variable degree of editing by ROs, depending on the automatic contouring method used. In this regard, AI methods are promising although they need to be instructed. Recently, computing power availability has promoted the development of AI automatic segmentation methods^16^, ^17^ and there are several commercially available AI segmentation modules: https://mirada‐medical.com/product/dlcexpert/, https://www.therapanacea.eu/our‐products#, https://www.mvision.ai/product/, https://www.mimsoftware.com/radiationoncology/contour_protege_ai.

In a previous work,^18^ we reported a pelvis CT atlas generation and optimization using a multi‐atlas approach implemented in MIM MAESTRO commercial software (MIM Software, Cleveland, OH, USA). We decided to create our atlas by optimizing both the choice of representative subject and sample size, as well as the registration and finalization algorithm, demonstrating that time and effort invested in atlas and workflow creation and optimization provides more reliable results.^18^

In the present retrospective study, we tested the potential benefits of introducing our optimized CT pelvis atlas in clinical practice. For this purpose, we involved five ROs and six previously treated test subjects, evaluating differences in contouring time and intra‐ and inter‐observer variability between manual (M) and automated segmentation approaches (AM).

To our knowledge this is the first study where the use of an optimized CT pelvis atlas has been evaluated in terms of contouring time, contouring accuracy and intra‐ and inter‐observer variability. Moreover, we believe that our clinical validation method could be useful in validating any kind of auto‐contouring system.

## 2 MATERIALS AND METHODS

A Brilliance Big Bore CT scanner (Philips Medical Systems, Cleveland, OH, USA) was used to acquire 120 kV CTs with 3 mm slice thickness, 600 mm field of view and 512 × 512 matrix. To automatically generate contours, we used the pelvis CT atlas previously created and optimized using the MIM MAESTRO (v.6.8.2) commercial framework.^18^ The software adopts a multi‐subject, atlas‐based segmentation method that enables users to select both *N* atlas subjects and one atlas representative subject, to create the atlas. For auto‐contouring, *k* (<*N*) best matching subjects are chosen automatically, based on image similarity criterion, and registered to the new subject to obtain *k* contours sets, then used for label fusion. The atlas is invoked by an automatic, customizable workflow. Our atlas was composed of 55 pelvis CT studies (manually segmented for radiotherapy planning), whose contours were thoroughly reviewed by a senior RO. In addition to the pelvis CT, bladder, rectum, femoral heads, and pelvic lymph‐nodes (PLN) contours were included in the atlas.

**FIGURE 1:**
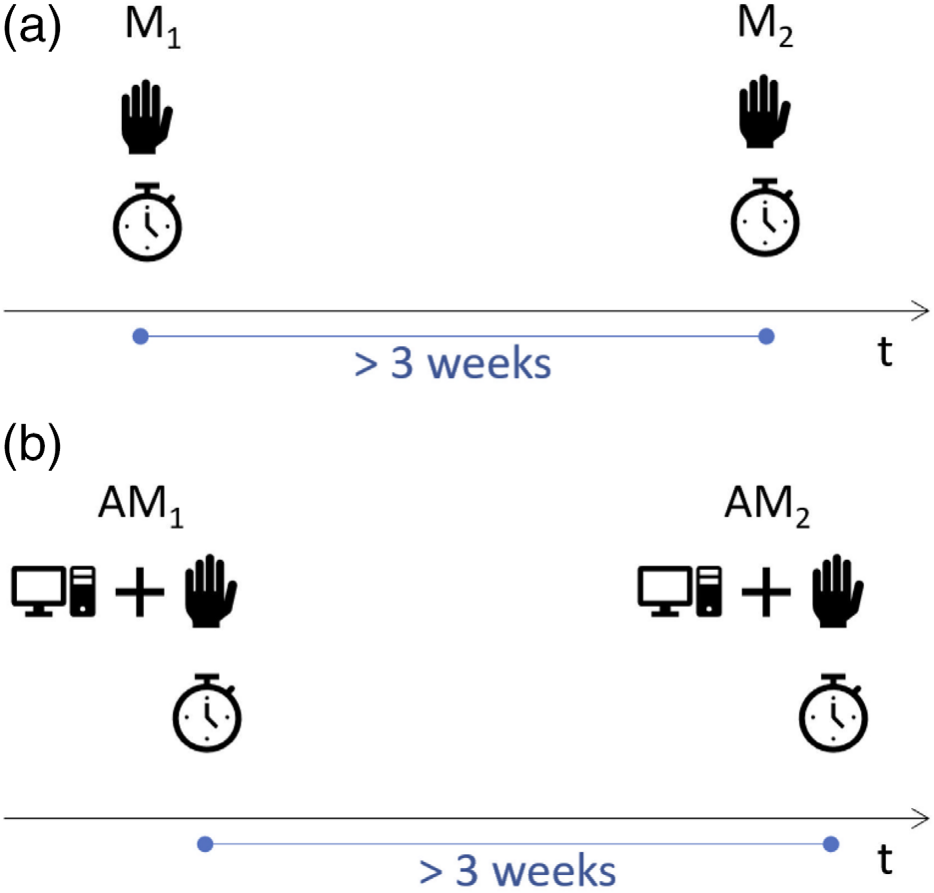
For each of the six test subjects, each region of interest (ROI) was manually contoured by each of the five radiation oncologists (ROs) two times, M1 and M2, with M2 at least 3 weeks after M1 (a). The same approach was used for AM1 and AM2 automatic contour editing (b). AM2 contours were actually produced several months after the other three groups of contours. For each contouring (M1, M2) or editing (AM1, AM2) contouring time was registered

In this retrospective study, the potential impact of introducing auto‐contouring into the clinical workflow to reduce intra‐ and inter‐observer variability and contouring time was investigated. Tests were based on 3 mm slice thickness CT studies of six previously treated test subjects, enrolling five senior (5–15 years experienced) ROs for re‐contouring and auto‐contour editing. For each test subject, bladder, rectum, PLN, and femoral heads were manually contoured by each RO, producing the manual 1 (M1) contour sets. The number of slices for each contour varied from 18 to 34, 35 to 43, 28 to 51, and 32 to 39 for bladder, rectum, PLNs, and each femoral head, respectively, depending on the test subject and RO. To evaluate intra‐observer variability, the same procedure was repeated to produce the manual 2 (M2) contour set, after a minimum of 3 weeks to minimize memory recall bias. In both cases, each RO recorded the contouring time for each test subject and structure (Figure 1a). Our CT pelvis atlas was then used to automatically generate contours of the same test subjects. Each RO reviewed and, if required, modified the auto‐contours noting the editing time for each structure and test subject. These automatically generated and, then, manually modified contours are called the automatic‐modified 1 (AM1) contour set.^18^ Again, after a minimum period of 3 weeks (actually several months), the same procedure was repeated to produce the automatic‐modified 2 (AM2) contour set (Figure 1b). Each contour set (M1, M2, AM1, AM2) comprises 150 structure contours (6 test patients × 5 ROs × 5 structures), thus a total of 600 contours were collected.

**FIGURE 2:**
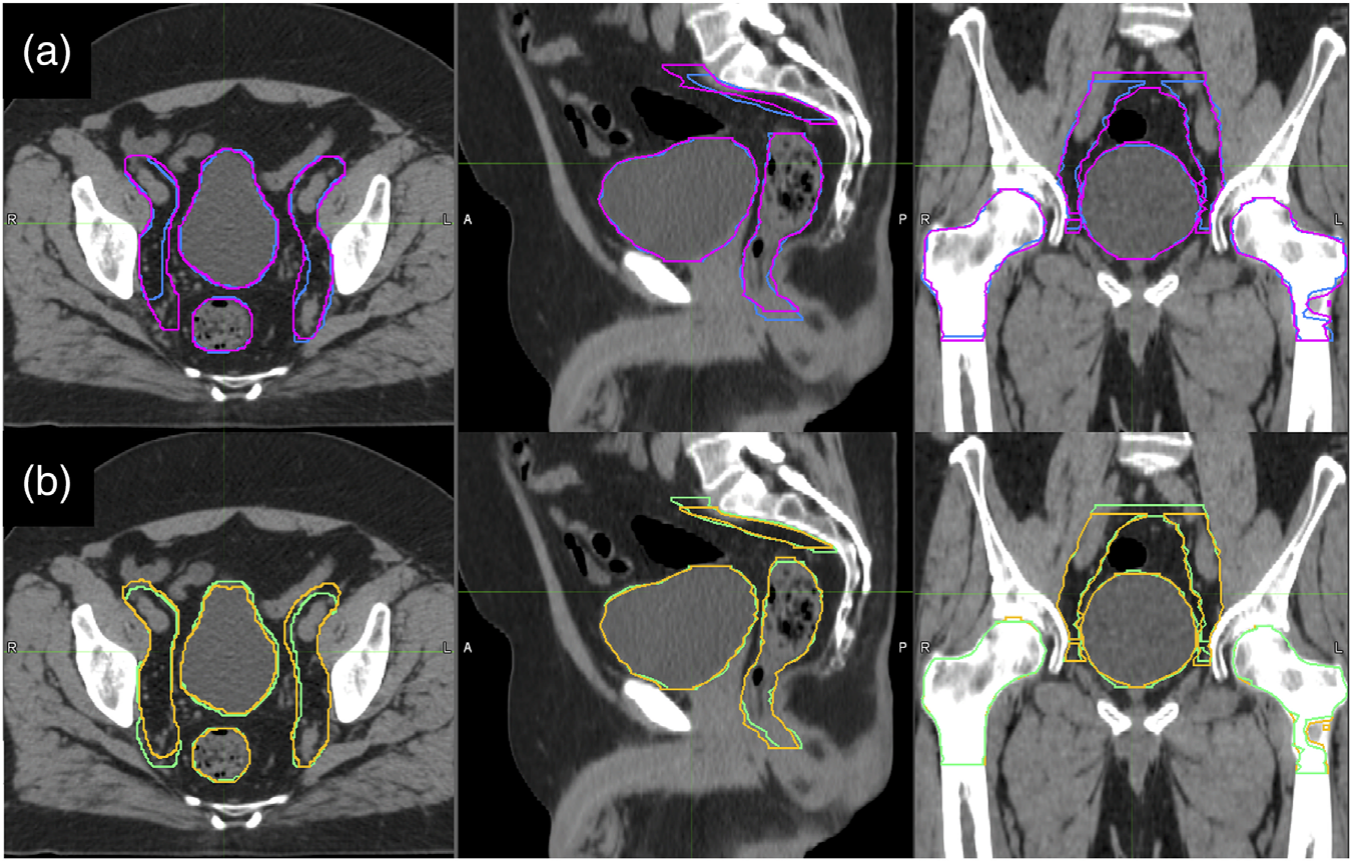
Single radiation oncologist (RO) contours are shown on the computed tomography (CT) images of a test patient: (a) M1 (blue) and M2 (magenta) contours; (b) AM1 (green) and AM2 (yellow) contours

In Figure 2, single RO contours are shown on the CT images of a test patient: Figure 2a shows M1 (blue) and M2 (magenta) contours, while Figure 2b shows AM1 (green) and AM2 (yellow) contours. Manual contouring and editing were performed with the Philips Pinnacle^3^ treatment planning system 2D painting tool.

Intra‐ and inter‐observer variability and time savings were evaluated as described in Sections 2.1–2.3. Finally, a statistical test, based on six test subjects and enrolling four ROs, was conducted to exclude any potential bias introduced by the automatic contours (Section 2.4).

### 2.1 Intra‐observer variability

To assess whether the atlas‐based contouring approach can help in reducing intra‐observer variability, we evaluated Dice similarity coefficient (DSC) and mean distance to agreement (MDA) scores between M1 and M2 contours and we compared these results with those obtained between AM1 and AM2.

**FIGURE 3:**
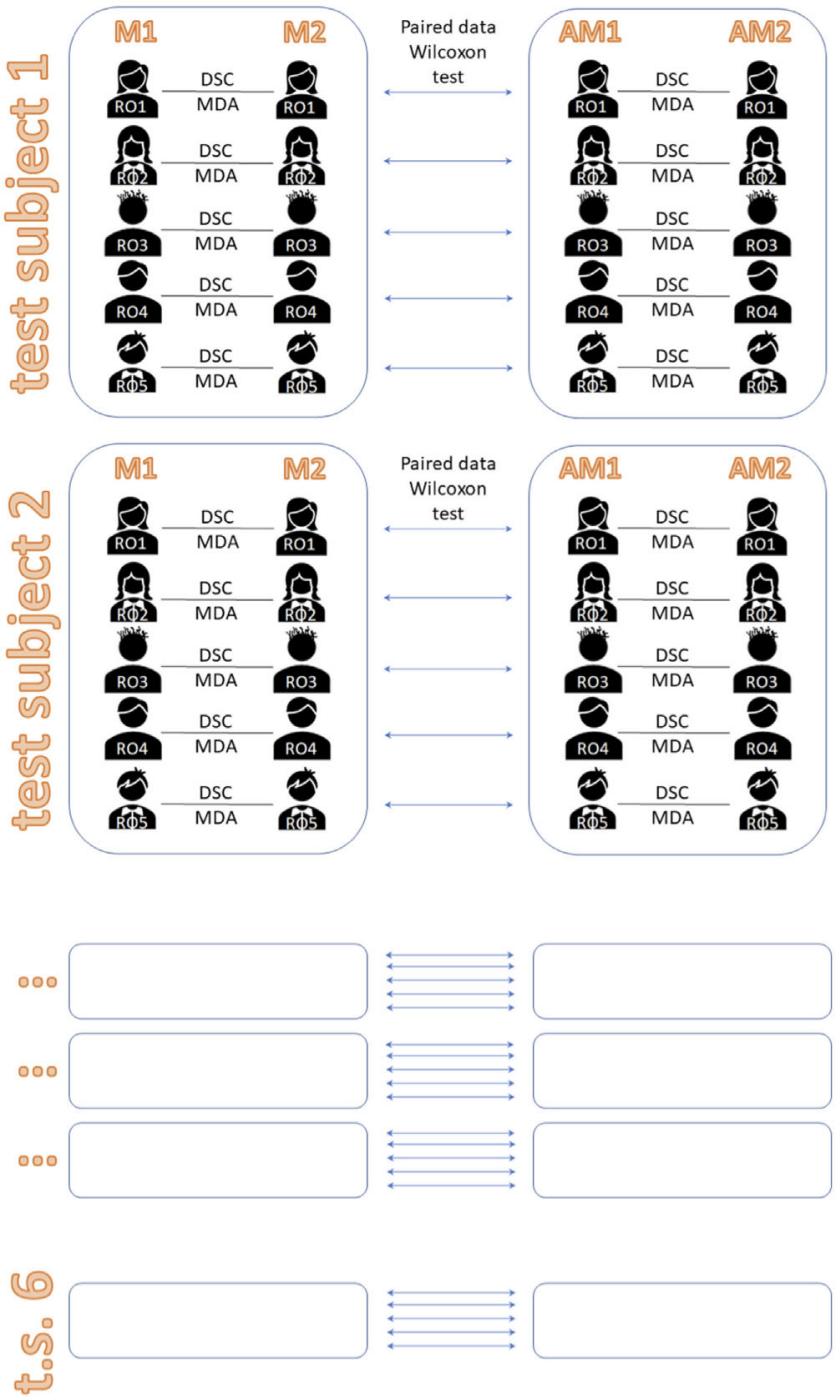
Intra‐observer variability. For each region of interest (ROI), contours delineated by each radiation oncologist (RO) at different times (M1 and M2, AM1 and AM2) were compared in terms of Dice similarity coefficient (DSC) and mean distance to agreement (MDA) indices for each of the six test subjects, thus obtaining two (M and AM) 30 dimensional (5 ROIs × 6 test patients) vectors, both for DSC and MDA. Wilcoxon signed‐rank statistical test was applied to compare intra‐observer variability of M and AM contours, both for DSC and MDA

Figure 3 illustrates how the comparison between M1 and M2 delineated by the same RO for each test subject generated 30 DSC and 30 MDA indices (5 ROs × 6 test subjects) for each structure. Similarly, a comparison of AM1 and AM2 contour sets generated 30 + 30 (DSC + MDA) couples of similarity indices. We decided to conduct our comparison using both DSC and MDA because, while DSC use is widely documented in the literature, it has the limitation of being dependent upon contour volume. Conversely, while MDA is not frequently reported, it does not present this limitation.

For each structure, we evaluated the statistical significance of differences between groups of indices obtained for M and AM contours with Wilcoxon signed‐rank test for paired data (30 + 30).

### 2.2 Inter‐observer variability

**FIGURE 4:**
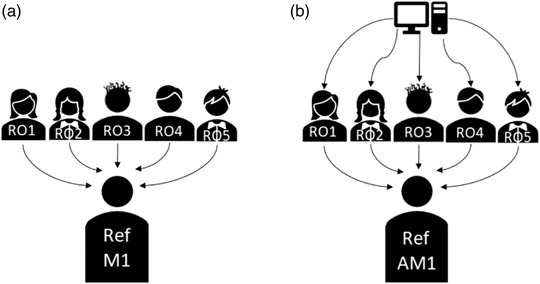
For each region of interest (ROI) of each test subject, M1 contours by the five radiation oncologists (ROs) were combined with STAPLE^28^ finalization algorithm to obtain a common reference Ref_M1_ (a). Similarly, AM1 contours (automatic contours, manually corrected by ROs) were combined to obtain Ref_AM1_ (b)

Assessing inter‐observer variability is always challenging, because of the difficulties in defining references. This problem can be addressed in different ways, for example, by performing two‐by‐two comparisons^20^, ^21^, ^22^ or by designating one set of contours as the reference.^19^, ^23^ In this study, we applied the STAPLE finalization algorithm^24^ to the contours outlined by all ROs, to define a reference set (Ref) used as the shared gold standard. This procedure was used on M1 and AM1 contours obtaining Ref_M1_ (Figure 4a) and Ref_AM1_ (Figure 4b), respectively. Ref_M2_ and Ref_AM2_ were obtained in the same manner.

The differences between each M1 and Ref_M1_ and each AM1 and Ref_AM1_ were evaluated using DSC and MDA.

**FIGURE 5:**
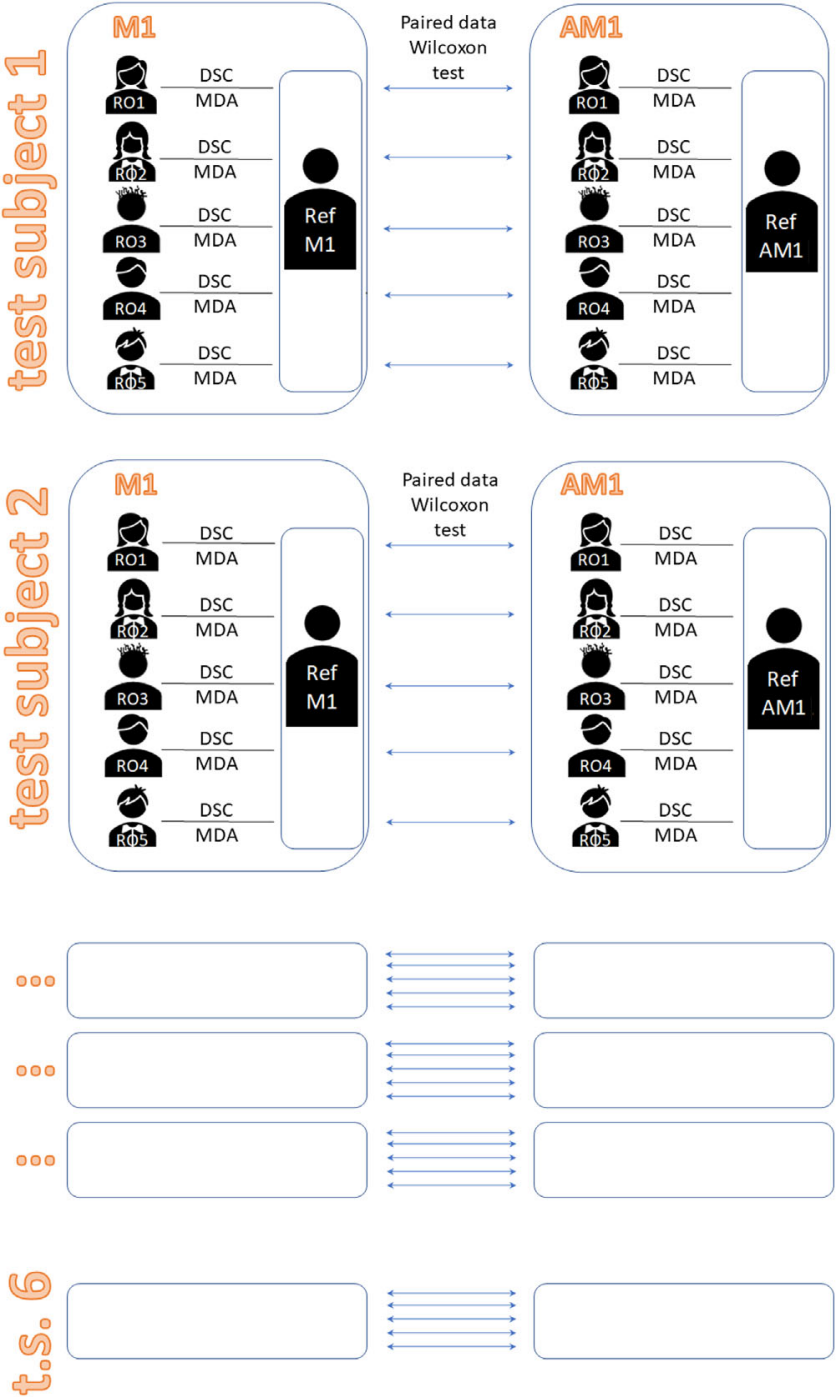
Inter‐observer variability. For each region of interest (ROI) of each test subject, contours M1 and AM1 by each radiation oncologist (RO) were compared to Ref_M1_ and Ref_AM1_, respectively, for both Dice similarity coefficient (DSC) and mean distance to agreement (MDA) indices. Thus, for each index (DCS, MDA), two (M1 and AM1) 30 dimensional (5 ROIs × 6 test patients) vectors of indices were obtained and compared with Wilcoxon signed‐rank test for paired data to evidence any possible effect on inter‐observer variability due to the introduction of automatism

For each structure, the comparison between M1 and Ref_M1_ for all ROs (five) and test subjects (six) generated 30 + 30 (DSC + MDA) similarity scores. The comparison between AM1 and Ref_AM1_ produced other two 30‐dimensional similarity score vectors. M2 and AM2 datasets were elaborated analogously. Comparison between (DSC + MDA) groups of scores for M and AM contour sets enabled the evaluation of intra‐observer variability for both methods (Figure 5).

### 2.3 Time savings

Subsequently, for each RO, we evaluated and compared average manual contouring and editing time for the whole pelvis. Data were averaged over six test subjects; M1 and M2 data were averaged to obtain an average manual contouring time (*T*_M_) for each RO. Similarly, the average time for editing automated contours *T*_AM_, was calculated by averaging AM1 and AM2 editing times. Time savings, defined as |*T*_M_ ‐ *T*_AM_|/*T*_M_, was evaluated for each operator.

The analysis was also performed for each structure, averaging over the five ROs and six test subjects. Then, the overall time savings, as an average across five ROs and six test subjects, was also calculated for the whole pelvis.

A more detailed analysis was done by creating boxplots of contouring and editing time: for each structure, four boxplots were created, containing M1, M2, AM1, and AM2 data. Each boxplot represents 30 time data (5 ROs × 6 test patients). Paired data (for the same test subject and RO) were compared using statistical tests.

### 2.4 Bias

Finally, to point out any possible bias introduced by automation, a blind test was performed by ROs. The test aimed to compare M and AM contours delineated by the same RO. To assure judgment impartiality, each RO was asked to examine a series of six contour sets containing both his/her own contours (M and AM) and four additional “mixed” sets (two M and two AM, randomly extracted from M1, M2, AM1, and AM2, thus including contours by other ROs). The purpose of adding “mixed” contours was to avoid judgment bias: the presence of other RO contours better‐ensured judgment objectivity. The test was blind since each RO did not know the operator who delineated the contours nor the contouring method. Since one RO was not available, we restricted the blind test to four operators.

For bladder, rectum, PLN, and femoral heads (considered together), each RO judged the acceptance (decision YES) or the opportunity/necessity to correct (decision NO) of every examined contour. The decision of confusing contours was not included in the statistical analysis. Acceptance rates of paired M and AM contours by the same RO were compared by means of a McNemar's statistical test^25^ (also known as paired or matched chi‐square) with 96 elements (4 regions of interest (ROIs) × 6 test subjects × 4 bias test ROs). The H0 hypothesis is that ROs are not influenced by the automatic contour generated by MIM software, so contour accuracy is not affected by any bias introduced in the preliminary automated contouring phase.

### 2.5 Statistical tests

Statistical tests for paired data were applied to compare M1 to M2, AM1 to AM2, or M to AM data, for the same RO and test subject. *t*‐Test or Wilcoxon signed‐rank test were used to statistically compare the location of DSC and MDA distributions (the former for normally distributed data and the latter for data not‐normally distributed). To guide the choice between parametric and non‐parametric statistical tests, a normality test of Shapiro–Wilk was conducted.

To our knowledge, there is no appropriate statistical test to compare dispersion of not‐normally distributed data, so we used the interquartile range (IQR) and we evaluated the IQR ratio between the two distributions.

Two‐tailed analyses were performed and a significance level of 0.05 was adopted. Online calculators were used to perform Shapiro–Wilk test (http://www.statskingdom.com/ and https://www.gigacalculator.com/calculators/normality‐test‐calculator.php) and Wilcoxon signed‐rank test (https://www.socscistatistics.com/).

## 3 RESULTS

### 3.1 Intra‐observer variability

**FIGURE 6:**
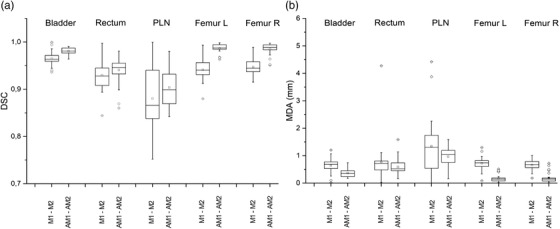
Intra‐observer variability. Boxplots for each structure of 30 Dice similarity coefficient (DSC) (a) and 30 mean distance to agreement (MDA) (b) similarity indexes between contours at two time points (1 and 2), calculated for the five radiation oncologists (ROs) and the six test subjects (5 ROs × 6 test subjects = 30 indices for each boxplot). On the *x*‐axis, manual and automatic procedures are indicated with the terms M1–M2 and AM1–AM2, respectively

For each structure, the DSC and MDA calculated for each test patient and each RO, between M1–M2 and AM1–AM2, were used to generate boxplots, shown in Figure 6a (DSC) and Figure 6b (MDA). Each boxplot represents 30 data points (6 test subjects × 5 ROs). In all the box‐whisker plots throughout this study, whiskers correspond to 5th and 95th percentile and odd data are plotted singularly. For both femurs, we found an important reduction of intra‐observer variability, supported by the significant Wilcoxon signed‐rank test result with *p* < 0.001 both in terms of DSC and MDA. Even for the bladder, we obtained *p* < 0.001 both for DSC (*t*‐test) and MDA (Wilcoxon signed‐rank test). On the contrary, for both rectum and PNL, we did not find any statistically significant difference neither in terms of DSC nor in terms of MDA.

To quantify the change in intra‐observer variability, we report the variation in terms of MDA 95th percentile for each structure from M to AM contours: 1.0 to 0.7 mm, 1.1 mm unaltered, 3.2 to 1.5 mm, 1.1 to 0.4 mm, and 1.0 to 0.6 mm for bladder, rectum, PLN, femoral head left, and femoral head right, respectively.

### 3.2 Inter‐observer variability

**FIGURE 7:**
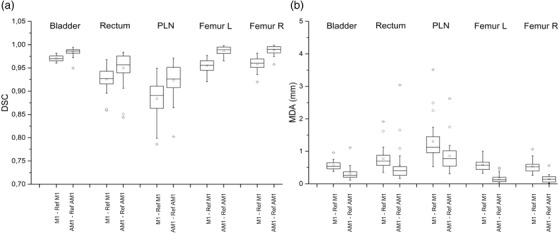
Inter‐observer variability. Boxplots of 30 Dice similarity coefficient (DSC) (a) and 30 mean distance to agreement (MDA) (b) similarity indexes for the five radiation oncologists (ROs) and the six test subjects were evaluated and placed side‐by‐side for manual (M1–Ref_M1_) and automated (AM1–Ref_AM1_) procedures and for each structure

The comparison between inter‐observer variability of manual procedure (M1 vs. Ref_M1_) and automated procedure (AM1 vs. Ref_AM1_) is shown in Figure 7, both in terms of DSC (Figure 7a) and MDA (Figure 7b). Each boxplot (one structure) is based on 30 points (5 ROs × 6 test subjects). For statistical analysis, M and AM indices are paired for the same RO and test subject. The improvement in accuracy (DSC increase and MDA decrease for AM1 with respect to M1) is statistically significant: *p* < 0.001 for all ROIs, both for DSC and MDA. The IQR ratio between AM and M contours is always ≤1, both for DSC and MDA (≤0.3 for femoral heads, DSC). Unfortunately, we did not find any statistical test to compare the dispersion of not‐normally distributed data, so we were not able to establish if the IQR decrease (AM vs. M) might be considered statistically significant. The analysis was repeated for M2 and AM2 contours, confirming the statistically significant reduction of inter‐observer variability (*p* < 0.001 for all ROIs, both for DSC and MDA).

**FIGURE 8:**
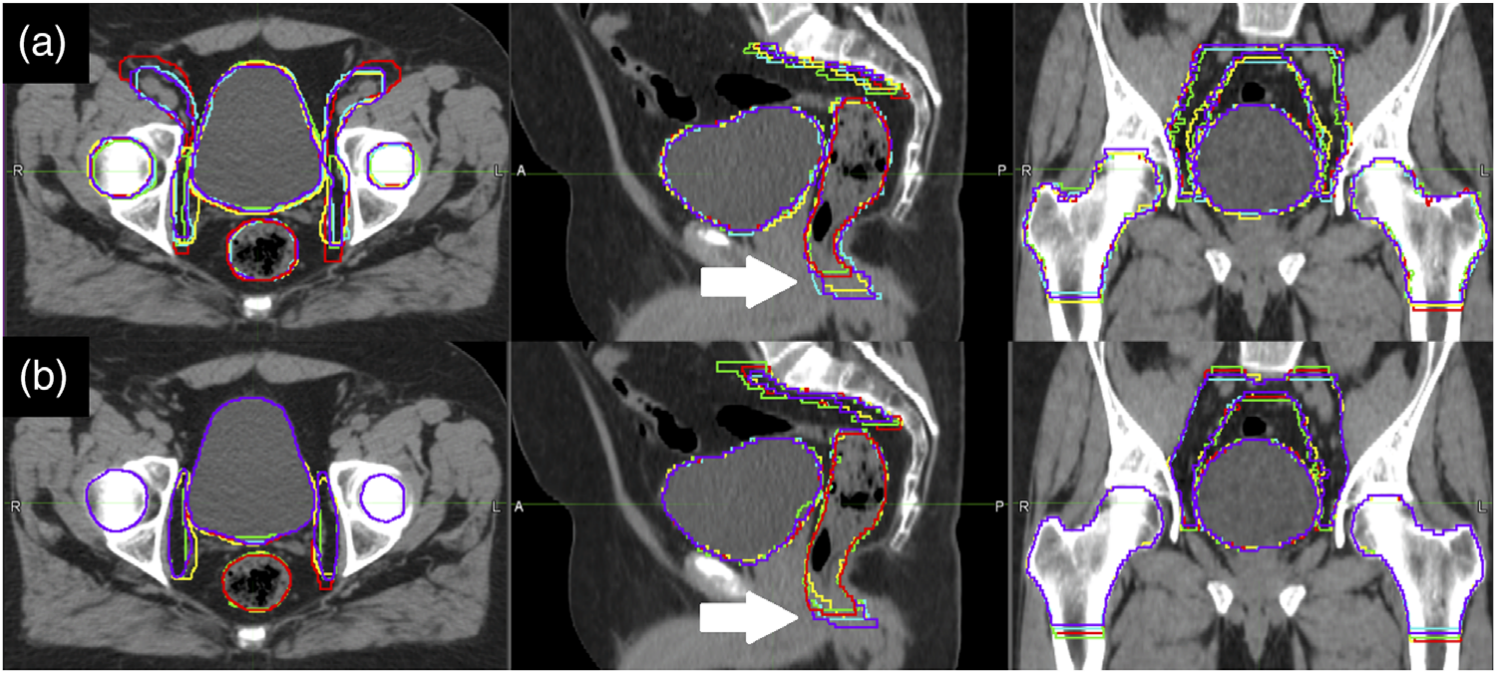
Manual M1 contours (a) and AM contours (b) are compared for one of the six test subjects

In Figure 8, M1 (Figure 8a) and AM1 contours (Figure 8b) of one representative test subject are presented. Each contour color corresponds to one of the five ROs. The reduction of inter‐observer variability in AM1 set can clearly be noted, for example, in the inferior limit of the rectum (indicated with the arrow). A critical review of delineated volumes revealed that the rectum inferior limit is the most critical issue influencing residual inter‐observer variability in AM contours.

### 3.3 Contouring time

**FIGURE 9:**
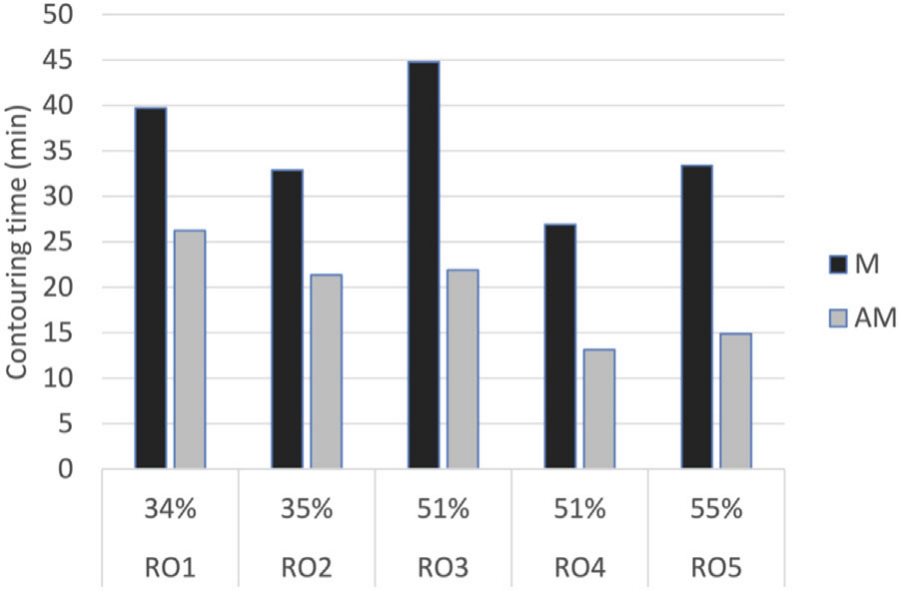
*T*_M_ and *T*_AM_ pelvis contouring time and percentage time savings, depending on radiation oncologist (RO). Values are averaged over six test subjects. *T*_M_: average time for manual contours, *T*_AM_: average time for editing automated contours

*T*_M_ and *T*_AM_ for the whole pelvis, averaged over all test subjects for each RO, are reported in Figure 9. The percentage of time savings between *T*_M_ and *T*_AM_ for each RO is indicated.

Table 1 shows the averaged *T*_M_ and *T*_AM_ over all ROs and test subjects with both absolute (*T*_M_ and *T*_AM_) and percentage of time savings for each structure and for the whole pelvis. Femoral heads have been treated as one single ROI for time analysis.

**TABLE 1:** *T*_M_, *T*_AM_, and percentage of time saving, for each structure and for the whole pelvis

	*T*_M_ (min)	*T*_AM_ (min)	|*T*_M_ − *T*_AM_| (min)	|*T*_M_ − *T*_AM_|/ *T*_M_
Rectum	4.0	2.7	1.3	32%
Bladder	4.2	2.6	1.6	39%
PLN	18.7	12	6.7	36%
Femoral heads	8.7	2.2	6.4	74%
Pelvis	35.5	19.5	16	45%

*Note*: Values are averaged over five radiation oncologists (ROs) and six test subjects. *T*_M_: average time for manual contours, *T*_AM_: average time for editing automated contours.

Abbreviation: PLN, pelvic lymph‐node.

Time savings percentage varies by ROs (from 34% to 55% and 12 to 23 min whole pelvis, respectively) and ROIs (from 1.3 min and 32% for rectum to 6.4 min and 74% for femoral heads). Average time savings (over all ROs and test subjects) for the whole pelvis was 45%.

**FIGURE 10:**
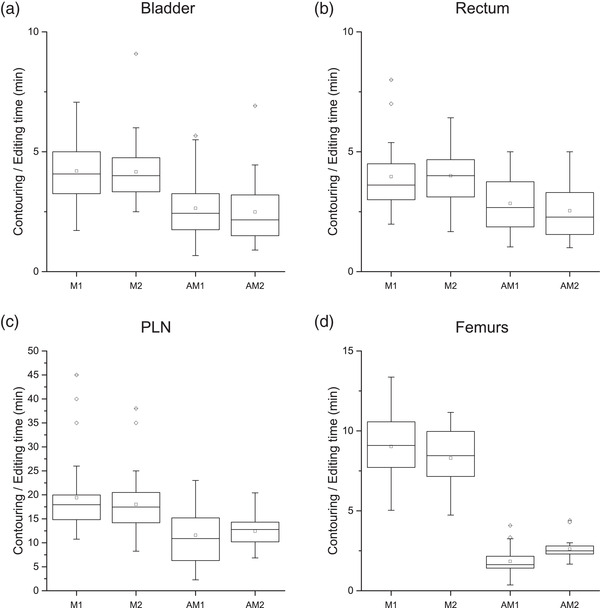
Contouring (M1 and M2) and editing (AM1 and AM2) times. For each structure, four boxplots were generated, for M1, M2, AM1, and AM2, respectively, each representing 30 data points (5 ROs × 6 test patients)

Figure 10 shows the boxplots of contouring (M1 and M2) and editing (AM1 and AM2) times for bladder (Figure 10a), rectum (Figure 10b), PLN (Figure 10c), and femoral heads (Figure 10d). Each boxplot series contains 30 data points (5 ROs × 6 test patients). After establishing the consistency between M1 and M2 contours and between AM1 and AM2 contours, M contouring times and AM editing times were compared for each ROI by *t*‐test analysis.

For each of the four ROIs, M1 was compared to M2 with a *t*‐test: the null hypothesis is that M1 and M2 are two samples extracted from the same data distribution. A second *t*‐test was conducted in the same manner to compare AM1 with AM2, for a total of eight tests (M1 vs. M2 and AM1 vs. AM2, for each of the four ROIs). Only the comparison between AM1 and AM2 for femoral heads presented statistically significant results (*p* < 0.05), favoring AM2. The other seven tests were not statistically significant.

All *t*‐tests for the comparison of M to AM paired data (M1–AM1 and M2–AM2 for each structure, for a total of eight tests) presented statistically significant results with *p* < 0.001.

### 3.4 Bias

Bias test results are reported in Table 2, where NO and YES decisions indicate that contours needed correction or were accepted, respectively.

**TABLE 2:** Contingency table for McNemar's test applied to radiation oncologist (RO) bias test to evidence any possible bias introduced by automatic contouring

AM	M	
	NO	YES
NO	3	4
YES	8	81

*Note*: Acceptance (score YES) or refusal (score NO) was expressed altogether over 96 structures, considering four radiation oncologists (ROs), four regions of interest (ROIs) (bladder, rectum, pelvic lymph‐node (PLN), and femoral heads), and six test subjects.

Abbreviations: M, manual contour; AM, automatic‐modified contour.

Table 2 shows that in eight (out of 96) cases, M was rejected, and AM accepted, while only in four (out of 96) cases the judgment was in favor of M, thus favoring AM contours sets. However, McNemar's test resulted in *p* = 0.39, indicating that differences between AM and M contours in terms of clinical accuracy were not statistically significant. This suggests that even if bias due to the editing of pre‐generated contours (AM) as opposed to manually contouring (M) from scratch was present, its effects were not detectable with this sample size (Nc = 96).

## 4 DISCUSSION

In radiotherapy planning clinical practice, the greater difficulty in the delineation of certain structures, such as PLN and rectum, translates into a greater intra‐observer variability, which could explain lower DSC and higher MDA values compared to the bladder and femoral heads (Figure 6).

With regard to data dispersion (range and IQR in the intra‐observer variability boxplots), we believe this could be related to inter‐observer variability (each boxplot contains data from all five ROs). This could explain the observed range and IQR decrease due to auto‐contouring for all ROIs (both DSC and MDA).

From a geometrical and statistical point of view, the AM contours showed a significant reduction in intra‐observer variability only for bladder and femoral heads. For rectum and PLN, the statistic test did not evidence a significant difference in intra‐observer variability. Nonetheless, the boxplots visually suggest a trend in favor of AM contours, which could perhaps lead to a significant result if a larger statistical sample was available; indeed, the indices distribution is more widespread for rectum and PLN than for bladder and femoral heads. This could be explained by noting that variability of delineation of some organs at risk (e.g., rectum) may be related to difficulties in detecting anatomical structures and determining their limits. For example, the lower limit of the rectum is usually determined by the cranial limit of the elevator ani muscle, which can be critical to define in planning CT (Figure 8), and the boundary between rectum and prostate could be difficult to identify because of poor contrast between the two structures. Intra‐observer variability of PNL volumes may be caused by uncertainty in pelvic nodal clinical target volume definition. Indeed, these structures are only partially defined by real anatomical boundaries such as muscles or bones, and the rest of the delineation relies on an arbitrary 7‐mm surrogate margin around blood vessels. Furthermore, vessels are more difficult to identify by use of CT images without contrast. Lack of intra‐observer reduction for structures such as rectum and PLN may thus be related to intrinsic delineation uncertainty (which affects both auto‐contours and manual contours), and consequently increased need for editing automatic contours.

From a clinical point of view, however, the reduction of the 95th percentile of MDA values was not relevant for any of the considered structures (from M to AM contours: 1.0 to 0.7 mm, 1.1 mm unaltered, 3.2 to 1.5 mm, 1.1 to 0.4 mm, and 1.0 to 0.6 mm for bladder, rectum, PLN, femoral head left, and femoral head right, respectively), even considering the slice thickness of 3 mm of our CT studies. Hence, from this study we can conclude that intra‐observer variability did not benefit significantly from introducing automatic contouring in the clinical workflow. A possible explanation is that the participating ROs were senior, experienced ROs. It could be interesting to repeat the study involving less experienced physicians. Furthermore, the differences in intra‐observer variability may be object of a further study investigating dose distribution and dose volume histogram.

Concerning the interpretation of inter‐observer variability, both data dispersion and position need to be considered. A reduction in inter‐observer variability results in a reduction of similarity indices dispersion and also affects data position. Indeed, since similarity indices are calculated for each single RO's contour as compared to the reference contour (obtained by combining the contours of all enrolled ROs), the higher similarity among each RO's contour, the more similar they will be to the common reference, and this will result in higher DSC values and lower MDA values.

It is worthwhile to discuss the number of ROs enrolled and the method used to define inter‐observer variability reference. Contours by *n* different operators can be compared pairwise^22^, ^23^, ^24^ (the comparisons number being determined by the pair combinations between operators, i.e. n!/(2·(n−2)!)). To reduce the computational effort when dealing with more than three observers, defining a common reference contour to which each observer's contour can be compared might be useful.^19^, ^23^ Joskowicz et al.,^26^ in their study about inter‐observer variability, concluded that “The variability in manual delineations for different structures and observers is large and spans a wide range across a variety of structures and pathologies. Two and even three observers may not be sufficient to establish the full range of inter‐observer variability”. Based on this conclusion, we decided to enroll five ROs. Since in this case the number of pairwise combinations would be 10 (which would lead to an important computational/elaboration effort), we decided to define a common reference by means of STAPLE finalization algorithm.^19^ It is important to point out that, due to this approach, inter‐observer variability is not expected to be on the same scale of intra‐observer variability. Indeed, based on our definition of intra‐ and inter‐observer variability, the former is like the deviation between two measured values, while the latter is more similar to the deviation of each measured value compared to the mean of the values obtained from repeated measurements of the same variable. In theory, we could expect a higher inter‐observer variability than intra‐observer variability, although this direct comparison can be done only with a pairwise, inter‐observer comparison and is not applicable to our study. However, based on our inter‐observer variability definition, DSC maximum values for inter‐observer variability could be higher than those of intra‐observer variability, even though the latter were evaluated on repeated contours of the same operator. For this reason, statistics of inter‐ and intra‐observer variability are not directly comparable, and we never reported them on the same plot. The definition of inter‐observer variability with respect to a common reference contour implies that DSC and MDA values have no absolute meaning. The only meaningful information is to test the statistical significance of the difference between M and AM data.

In our retrospective study, DSC and MDA distributions for manual and automated contouring approaches demonstrated that the introduction of automated contouring into the clinical workflow could reduce inter‐observer variability. Five ROs and six previously treated subjects were involved in this study and for all evaluated structures (bladder, rectum, PLN, and femoral heads) a statistically significant (*p* < 0.001) difference in the contours’ variability between manual and automated approaches was observed.

This aspect has been investigated in other studies^5^, ^19^, ^22^, ^23^ but only two of these^5^, ^22^ report about auto‐contouring of pelvis region. Langmack et al.^5^ enrolled one dosimetrist who was asked to contour the pelvis CT studies of eight test subjects from scratch and edit automatic contours of the same test subjects. Both contours were compared with manually depicted contours by one RO (acting as gold standard) to quantify possible improvement through the introduction of automated contouring. A statistically significant improvement was assessed only for prostate contour.

Young et al.^22^ enrolled three ROs for manual contouring and automated contours editing of endometrial cancer nodal volumes of 10 test subjects. Inter‐observer variability was evaluated by comparing contours of two ROs at a time. Only in one case out of three possible couples was inter‐observer variability significantly reduced statistically (*p* = 0.02), thanks to automation introduction in contouring.

To our knowledge, this is the first study where the use of an optimized CT pelvis atlas has been evaluated in terms of contouring time, contouring accuracy, and intra‐ and inter‐observer variability.

An average contouring‐time savings of 45% (36 min vs. 19 min) for the five structures considered (averaged over six test subjects, five test ROs) is the most appealing result of our study, with a statistically significant (*p* < 0.001) time saving for all structures. The degree of time saving is heavily dependent on the operator's clinical experience and familiarity with the atlas‐based segmentation tool and varies between 34% and 55% (Figure 9). This finding is compatible with results reported in previous studies.^1^, ^2^, ^3^, ^4^, ^5^ In general, contouring and editing time may be inherently biased because each physician saw the same patient CT four times and could have become familiar with each case. Nonetheless, the statistical analysis of time data for each ROI, shows a strong consistency between M1 and M2, as well as between AM1 and AM2. The only statistically significant result was found in the case of femoral heads, between AM1 and AM2 editing times (on average 1.84 and 2.61 min, respectively). Despite statistical significance, this difference cannot be considered clinically relevant, being a difference of only 46 s. On the other hand, the comparison between AM and M was statistically significant (*p* < 0.001) in favor of AM contours, in all cases (M1–AM1, M2–AM2, for all structures).

From a clinical point of view, saving time is advantageous only if the contouring remains robust and reliable, otherwise it is not easily applicable. In our study, the blind test to investigate the possible bias introduced by automated contouring, did not evidence any statistically significant difference between AM and M contours. The test was based on a statistical sample of 96 contours, obtained by enrolling four experienced ROs and considering six test subjects and four structures (bladder, rectum, femoral heads together, and PLN). As far as we know, this kind of systematic evaluation has never been reported before, although several studies have mentioned clinical validation of an automatic contouring method for pelvis CT.^1^, ^2^, ^3^, ^4^, ^5^, ^7^, ^14^

Based on these encouraging results in terms of time savings and increased reproducibility of our work, further investigation of these aspects by increasing the number of test ROs and test subjects might be desirable, to increase the statistical analysis power.

Of note, DSC of the automatic contours generated by our atlas and used for editing in this study is quite high compared to the results reported in the literature. In our previous work,^18^ we compared our results (mean DSC) for bladder and rectum with DSC values obtained from 19 other studies (DSC min, DSC max): 0.89 compared to (0.59, 0.95) and 0.83 compared to (0.47, 0.92) for bladder and rectum, respectively. As for femoral heads, we obtained a mean DSC of 0.96, compared to (0.69, 0.98), from 11 other studies. The share of studies that reported lower DSC values compared to ours was 13/19 (bladder), 15/19 (rectum), 9/11 (femurs). For pelvic nodes, we found only other two studies which reported mean DSC 0.74^27^ and 0.71,^28^ while we obtained a mean DSC of 0.85. Obviously, DSC values depend on the reference and cannot be considered an absolute measure of contour accuracy. However, this comparison reinforces our hypothesis that each clinic institution could benefit from a thorough optimization and even more customization of their own auto‐contouring atlas. Indeed, the aim of atlas customization is precisely to obtain contours as similar as possible to the manual reference contours, following the same contouring criteria which have been adopted in the considered institution. To obtain maximum benefit from auto‐contouring, the manual contouring accuracy of atlas subjects is a crucial element. As in clinical practice, adherence to guidelines for contouring is highly recommended. In this regard, a preliminary audit performed by the five ROs was able to evidence some minor differences in the contouring criteria and was, in the end, useful in reaching a higher degree of consensus among the ROs of our institution, before reviewing the atlas subjects’ contours and initiating the clinical validation reported in this work.

## 5 CONCLUSION

In this study involving five experienced ROs and six test subjects, a CT pelvis atlas and relative auto‐contouring workflow previously developed and optimized^22^ were retrospectively validated from a clinical perspective. Intra‐ and inter‐observer variability evaluated on AM contours were compared to those evaluated on M contours. Intra‐observer variability was significantly reduced for bladder and femoral heads, while no impact on rectum and PNL was obtained. Inter‐observer variability was reduced as well. The statistical analysis conducted on DSC and MDA data, referred to a common reference contour, demonstrated a statistically significant reduction of variability. The time savings achievable with auto‐contouring are operator‐dependent but an average reduction of pelvis contouring time of 45% is a promising result. Finally, we did not detect any bias introduced by automation.

The creation and customization of an atlas‐based workflow is a time‐consuming task that requires a careful selection of atlas subjects, a strict standardization of contouring criteria and a thorough tuning of atlas and workflow parameters, but our result could encourage institutions to implement their own local atlas‐based workflows.

## FUNDING INFORMATION

No funding was received for conducting this study.

## CONFLICT OF INTEREST

The authors have no relevant financial or non‐financial interests to disclose.

## CODE AVAILABILITY STATEMENT

MIM Maestro was provided to Azienda Ospedaliero Universitaria Careggi from Temasinergie italian distributor. The atlas contains sensitive data and we are therefore unable to share it. The customized workflow is available upon request: mail to marta.casati@unifi.it

## AUTHOR CONTRIBUTIONS

Stefania Pallotta devised the project. Marta Casati contributed to the study conception and design. Marta Casati and Stefano Piffer did material preparation, data collection, and analysis. All authors contributed to the results discussion. Marta Casati and Stefano Piffer wrote the first draft of the manuscript. Stefania Pallotta contributed with a first revision and all authors commented and contributed to obtain the last revision of the manuscript.

## Data Availability

Computed tomography studies were selected among patients treated in Azienda Ospedaliero Universitaria Careggi.
